# Quantification of Spent Coffee Ground Extracts by Roast and Brew Method, and Their Utility in a Green Synthesis of Gold and Silver Nanoparticles

**DOI:** 10.3390/molecules27165124

**Published:** 2022-08-11

**Authors:** Brian G. Yust, Niny Z. Rao, Evan T. Schwarzmann, Madisyn H. Peoples

**Affiliations:** 1 Department of Physics, Thomas Jefferson University, East Falls Campus, Philadelphia, PA 19144, USA; 2 Department of Chemistry and Biochemistry, Thomas Jefferson University, East Falls Campus, Philadelphia, PA 19144, USA; 3 College of Computing & Informatics, Drexel University, 3675 Market St., Philadelphia, PA 19144, USA

**Keywords:** green synthesis, nanoparticles, spent coffee grounds, byproducts, antioxidants, chemical analysis

## Abstract

Nanotechnology has become increasingly important in modern society, and nanoparticles are routinely used in many areas of technology, industry, and commercial products. Many species of nanoparticle (NP) are typically synthesized using toxic or hazardous chemicals, making these methods less environmentally friendly. Consequently, there has been growing interest in green synthesis methods, which avoid unnecessary exposure to toxic chemicals and reduce harmful waste. Synthesis methods which utilize food waste products are particularly attractive because they add value and a secondary use for material which would otherwise be disposed of. Here, we show that spent coffee grounds (SCGs) that have already been used once in coffee brewing can be easily used to synthesize gold and silver NPs. SCGs derived from medium and dark roasts of the same bean source were acquired after brewing coffee by hot brew, cold brew, and espresso techniques. The total antioxidant activity (TAC) and total caffeoylquinic acid (CQA) of the aqueous SCG extracts were investigated, showing that hot brew SCGs had the highest CQA and TAC levels, while espresso SCGs had the lowest. SCG extract proved effective as a reducing agent in synthesizing gold and silver NPs regardless of roast or initial brew method.

## 1. Introduction

Despite the socio-economic impact of the COVID-19 pandemic, coffee consumption and popularity continue to rise. Global coffee consumption saw an average compound annual growth rate (CAGR) of 1% from the 2017/2018 coffee year to the 2020/2021 coffee year, where a coffee year runs from October to the following September [1]. Regionally, Africa and the Middle East experienced the greatest increase in the consumption of coffee, promoting industry growth in those regions. Within the United States, weekly consumption of coffee increased to 73% of polled individuals in January 2022 from 69% in July of 2021, due to increased specialty coffee consumption [2]. Several popularized brewing methods, such as espresso and cold brew techniques, fall under this increase. Increased coffee consumption correlates to a rise in waste byproducts that have been repurposed in other industries, such as agriculture and biofuel, to follow better green chemistry practices.

Coffee bean extract has been analyzed and researched extensively, with particular emphasis on how consumption impacts health. Caffeine and chlorogenic acid derivatives are the primary biologically active molecules found within coffee, though coffee may be comprised of approximately 1000 other compounds, including phenolic compounds, diterpenes, methylxanthines, and melanoidins [3]. Chlorogenic acid derivatives have demonstrated positive protective activities, including anticarcinogenic, cardioprotective, anti-obesity, and antidiabetic effects [4]. Antioxidant activity contributes to these protective effects by alleviating the free radical imbalance within the body, thus reducing oxidative stress. Oxidative stress has been linked to neurological disorders such as Alzheimer’s disease and amyotrophic lateral sclerosis (ALS), as well as cancer, rheumatoid arthritis, and diseases of the cardiovascular, respiratory, and renal systems [5]. Beyond health benefits, coffee bean extract has been utilized in the synthesis of a variety of nanoparticles (NPs), including silver [6,7,8], gold [9], platinum, palladium [8], and copper [10,11]. Additionally, certain byproducts of the coffee extract manufacturing process, such as spent coffee grounds and coffee plant leaves, have been utilized in the synthesis of silver [12] and zinc oxide NPs [13], respectively. Research into NPs is continuously expanding due to their unique properties and wide range of applications.

NPs are known to exhibit interesting properties that can vary with characteristics such as size and morphology. Metal NPs have various applications in catalysis, optics, biotechnology, molecular sensing, targeted drug-delivery, and antimicrobial materials. In particular, silver, gold, and copper are among the most commonly synthesized NPs, due to their stability and variety of uses [14]. NPs can be synthesized into many different shapes and sizes, leading to different functionalities and applications. The surface plasmon resonance (SPR) absorption, which is caused by the resonant oscillation of electrons in the conduction band of these particles [15], also shifts based on the varying structure of metal NPs, allowing researchers to characterize these particles on the nano-scale through optical properties.

Many NPs are typically synthesized using techniques that utilize toxic or hazardous chemicals, which makes these synthesis methods less environmentally friendly. In order to minimize risk from exposure to toxic chemicals and reduce harmful waste, green synthesis methods are becoming more popular in the field of nanotechnology. The increasing demand for nanomaterials in a growing set of applications further requires minimizing the hazardous waste and maximizing the efficacy of green synthesis methods [16]. Green synthesized Au and Ag NPs are known to have many biological and medical applications. Both species of NP can be easily functionalized with antibodies, drugs, polymers, and amino acids [17,18,19,20]. Au NPs have been utilized in cancer diagnosis and therapy, photothermal therapy, biomedical imaging, targeted drug delivery, and enzyme inhibition [21,22,23,24,25,26,27]. Ag NPs have been utilized extensively as antimicrobial agents in surface coatings, wound dressings, catheters, textiles, and consumer products [14,28,29,30,31,32,33,34,35]. Towards achieving greener chemistry methods, synthesis of many different species of NP have been demonstrated using coffee liquid, coffee bean extract, or coffee grounds as a replacement for more caustic, synthesized chemicals such as sodium borohydride. These include silver [6,7,8], gold [9], platinum, palladium [8], copper [10,11], zinc oxide [36], selenium [9], alumina [37], and carbon dots [38]. Most of these syntheses utilize naturally available acids within the coffee product to reduce metal ions in solution and promote aggregation and growth into NPs. Coffee beans, coffee grounds, and coffee liquid are known to contain a host of bioactive compounds, including caffeine, chlorogenic acids, phenolics, and melanoidins. Of these, chlorogenic acid has been singled out and shown to be effective in the synthesis of silver [39], gold [40], platinum [41], and bimetallic NPs [42].

Like many food processing methods, coffee bean extraction processes leave behind a host of active molecules and compounds in the byproducts. The discovery of a diverse array of uses for these coffee byproducts resulted in an expansion of scientific research into coffee chemistry. Primary coffee byproducts include the coffee husk, pulp, silverskin, and spent coffee grounds (SCGs), each with a unique chemical composition [43,44]. SCGs are brewed coffee grounds that contain unextracted material in which active compounds are present. A variety of factors, such as extraction process and roasting temperature, impact the compound extraction yield and the composition of the extract [45,46]. Current research identifies the capacity of SCGs as a potential source of biofuel [47,48,49,50,51], antioxidants [43,44,52,53,54,55,56,57,58,59], flavonoids [60,61,62,63], sugars [64,65,66,67,68], and fertilizer [69,70,71,72], to name a few. Due to similarities in composition to unbrewed coffee grounds, SCGs should have related, unrealized applications in NP synthesis in line with coffee liquid, coffee bean extract, and unbrewed coffee grounds [73,74]. Utilization of coffee byproducts offers green chemistry, low-waste processes, and value-added propositions.

Motivated by the expansion of research into coffee byproducts, this study characterized the contents of SCG samples to quantify total caffeoylquinic acid (CQA), total antioxidant capacities (TACs), and efficacy in synthesizing silver and gold NPs. The rich antioxidant content of SCGs is well-documented [43,52,54,56,57,58,75,76,77]. However, the impact of factors such as brewing method [52,58,59] and degree of roast [53,58] on TAC levels in SCGs is not well-understood. This work estimated TAC levels in the aqueous extracts of six SCG samples (Table 1) from various degrees of roast and brewing methods using ABTS^+^ ((2,2′-Azino-bis(3-ethylbenzothiazoline-6-sulfonic acid) diammonium salt) decolorization assay, DPPH (2,2-Diphenyl-1-picrylhydrazyl) decolorization assays, Folin-Ciocalteu assay for total phenolic content (TPC), and ferric ion reducing antioxidant power (FRAP). This work also studied the efficacy of SCG extracts in the synthesis of two types of metal NPs, silver and gold, through a benchtop, green chemistry, single-pot method. Table 1 explains the abbreviations used to differentiate SCG samples among the multiple roast levels and brewing techniques compared in this work. The brewing methods employed were conducted to mimic standard home-brewing conditions with simplicity for industrial scaling.

## 2. Materials and Methods

### 2.1. Materials

Standards of 5-Caffeoylquinic acid (5-CQA, CAS: 327-97-9), Caffeine (CAS: 58-08-02), ABTS+• (2,2-Azinobis(3-ethylbenzothiazoline-6-sulfonic acid) diammonium salt) (CAS: 30931-67-0), Trolox (6-hydroxy-2,5,7,8-tetramethylchroman-2-carboxylic acid) (CAS: 53188-07-1), Potassium Persulfate (CAS: 7727-21-1), Sodium Acetate Trihydrate (CAS: 6131-90-4), Glacial Acetic Acid (CAS: 64-19-7), Methanol (67-56-1), Iron (II) Sulfate Heptahydrate (7782-63-0), TPTZ (2,4,6-Tris(2-pyridyl)-s-triazine) (CAS: 3682-35-7), Folin-Ciocalteu Reagent (MDL: MFCD00132625), Sodium Carbonate (CAS: 497-19-8), and Standardized 1 M Sodium Hydroxide (CAS: 1310-73-2) were purchased from Sigma-Aldrich (Milwaukee, WI, USA). DPPH (2,2-diphenyl-1-picrylhydrazyl) (CAS: 1898-66-4), Iron (III) Chloride Hexahydrate (CAS: 10025-77-1), and Gallic Acid (3,4,5-trihydroxybenzoic acid) (CAS: 149-91-7) were obtained from Alfa Aesar (Ward Hill, MA, USA), while Hydrochloric Acid (CAS: 7647-01-0), Ascorbic Acid (CAS: 50-81-7), Sodium Nitrite (CAS: 7632-00-0), and Aluminium Chloride Hexahydrate (CAS: 7784-13-6) were obtained from Thermo Scientific/Fischer Chemical (Nazareth, PA, USA). HPLC-grade Rutin Hydrate was acquired from Tokyo Chemical Industry (Tokyo, Japan). For NP synthesis, gold chloride hydrate (CAS 27988-77-8) was obtained from Sigma-Aldrich (Milwaukee, WI, USA), and silver nitrate (CAS 7761-88-8) was obtained from GFS Chemicals (Columbus, OH, USA).

### 2.2. Coffee Bean Preparation

Unroasted organic Colombian coffee beans were purchased from Golden Valley Coffee Roasters located in West Chester, PA, USA. Samples of 250 g of green coffee beans were roasted in a Hottop coffee roaster (Model No. KN-8828B-2K, HotTop, Hottop Coffee Roaster, Cranston, RI, USA) using the manufacturer’s default temperature-time setting. Beans were ejected from the roaster at two different final temperatures: 194 °C and 209 °C for medium and dark roasts, respectively. One batch was produced for each roast. Roasts were frozen for a minimum of 12 h [78] and ground using a conical burr coffee grinder (Model No. 560.01, Capresso, Montvale, NJ, USA) at the highest medium coarseness setting as designated by the manufacturer. The grinds were then sieved to retain particles in increments of <500 µm, 500–710 µm, and 710–1000 µm. Three sieves were used with mesh openings of 500 µm, 710 µm, and 1000 µm. Sieved grounds were stored at −18 °C before brewing.

### 2.3. Initial Coffee Brewing

Three brewing methods were prepared for a medium and dark roast Colombian coffee bean: cold brew, hot brew, and espresso. Brewing was completed for each roast level separately.

Cold-brew coffee was prepared in a French press using 10 g of ground coffee with a particle size range of 710–1000 µm in 100 g of room temperature deionized (DI) water. The mixture was steeped at room temperature for 24 h before the plunger was pressed into the grounds and coffee was poured off. SCGs were stored in an air-tight container at −18 °C.

Hot brew coffee was prepared in a French press by pouring 100 g of 100 °C DI water over 10 g of ground coffee of particle size 710–1000 µm. After 6 min, the plunger was pressed into the grounds and the coffee was poured off. SCGs were stored in an air-tight container at −18 °C.

Espresso coffee was prepared using a Breville ESP8SXL espresso machine filled with 75 mL of DI water poured over 10 g of coffee grounds of particle size < 500 µm. Spent espresso grounds were extracted and stored in an air-tight container at −18 °C.

### 2.4. Spent Coffee Extract Preparation

The previously stored SCGs were dried separately in an oven at 65 °C for 24 h to evaporate excess moisture. For brewing, a ratio of 1 part SCGs by weight to 5 parts boiling DI water by weight was used. The mixture was steeped for 5 min and filtered using the Hario V60 paper filter. Extracts were stored at 4 °C until analysis.

### 2.5. HPLC Analysis

Standard solutions and coffee extracts were analyzed using a methodology adapted from GL Sciences Technical Note No. 67 [62]. Analyses were performed on an Agilent 1200 Series high-performance liquid chromatography (HPLC) system fitted with a Supelco C-18, 5 µm column (15 cm × 4.6 cm) (Supelco, Bellefonte, PA, USA), and a C-18 guard column at 25 °C with a mobile phase mixture of 75% mobile phase A and 25% mobile phase B (A: 95% 2.0 mM phosphoric acid and 5% methanol; B: 95% methanol and 5% 2.0 mM phosphoric acid). The mobile phase flow rate was set at 1.0 mL/min with an injection volume of 10.0 µL. CQA isomers were detected using a diode array detector at 325 nm and 280 nm, respectively. Concentrations of 5-CQA were quantified via standard calibration curves. The retention time of 3-CQA and 4-CQA isomers was determined using standard solutions and quantitation of these two isomers was accomplished using the area of the 5-CQA standard combined with the respective molar extinction coefficients of the other two isomers as reported previously [76,79,80,81]. Two batches of coffee were brewed for each brewing style, and each batch of brewed coffee was analyzed in triplicate (n = 6).

### 2.6. Total Antioxidant Capacity Measurements

TAC of SCG extracts was estimated using four different methods.

#### 2.6.1. ABTS Assay

The ABTS^+^ radical cation decolorization assay method was previously described by Rao et al. [82]. Equal parts of 7 mM ABTS and 2.45 mM potassium persulfate solutions were combined and incubated for 16 h in a dark room to form a stock solution. A working solution was prepared by diluting the stock ABTS solution with DI water to obtain an absorbance within the range of 0.8 to 0.9 at 734 nm. An amount of 5 µL of sample was added to 3000 µL of the working solution. The resulting solution was mixed for 1 min and incubated for 6 min at room temperature. The absorbance of the solution was then measured at 734 nm using a Thermo Scientific Evolution 201 spectrophotometer. Trolox was used as a standard and results were expressed in mmol of Trolox equivalence (TE) per liter of extract. Each experiment was repeated in duplicate, and each sample was analyzed in triplicate (n = 6).

#### 2.6.2. DPPH Assay

The DPPH radical scavenging activity assay method was adopted from Odžaković et al. [83] and Muzykievicz et al. [84]. In brief, a working solution was prepared by diluting 0.3 mM DPPH methanolic solution with methanol to obtain an absorbance 1.000 ± 0.020 at 517 nm. A 5 µL aliquot of the SCG extract was added to 2850 µL of the DPPH working solution and vortexed for 1 min using a VWR Vortexer 2. The solution was allowed to incubate for 30 min in a dark room at room temperature before its absorbance was taken at 517 nm. Trolox was used as a standard, and results were expressed in mmol of Trolox equivalence (TE) per liter of extract. Each experiment was repeated in duplicate, and each sample was analyzed in triplicate (n = 6).

#### 2.6.3. Folin-Ciocalteu Assay (TPC)

Total phenolic content (TPC) was measured using the Folin-Ciocalteu method adopted from [84]. An amount of 10% *v/v* Folin-Ciocalteu reagent and 5 mM Na_2_CO_3_ aqueous solutions were prepared. Then, 2700 µL of 5 mM Na_2_CO_3_, 150 µL of 10% Folin-Ciocalteu, and 30 µL of SCG extract were mixed and incubated for 30 min in a dark room. After 30 min, sample absorbance was measured at 750 nm using UV-VIS spectroscopy. Gallic acid was used as standard and results were expressed in mg gallic acid equivalence (GAE) per liter of extract. Each experiment was repeated in duplicate, and each sample was analyzed in triplicate (n = 6).

#### 2.6.4. Ferric Ion Reducing Antioxidant Power (FRAP)

The reducing capacity of spent coffee brews was determined using the ferric ion reducing antioxidant power (FRAP) assay adapted from Muzykievicz et al. [84] and Benzie and Strain [85]. A FRAP working solution was prepared by mixing 1 part 10 mM TPTZ in 40 mM HCl, 1 part 20 mM iron (III) chloride hexahydrate, and 10 parts 300 mM acetate buffer of pH 3.6. Acetate buffer was prepared using 3.1 g sodium acetate trihydrate and 16 mL glacial acetic acid into a 1 L volumetric flask and filled with DI water. In a vial, 3 mL of working solution was mixed with a 100 µL sample (10 µL SCG extract + 90 µL DI water). The absorbance was measured at 593 nm after 15 min of incubation. Iron (II) sulfate heptahydrate and ascorbic acid were used as standards with results expressed in mg FeSO_4_ per liter of extract. Each experiment was repeated in duplicate, and each sample was analyzed in triplicate (n = 6).

#### 2.6.5. Silver and Gold Nanoparticle Synthesis and Preparation

The synthesis of silver and gold NPs followed a typical wet-bench chemical reduction method [86,87,88] with minor modifications detailed herein. To make this synthesis a low-waste process, NPs were synthesized using lab waste-products as reducing, nucleating, or capping agents to investigate their efficacy in replacing the more caustic reagents typically utilized. Specifically, SCG extracts were tested in this work. The NP synthesis was carried out in the presence of ambient white light. In a typical synthesis, a 50 mL Erlenmeyer flask equipped with a thermometer and aluminum foil cap was filled with 30 mL of DI water and placed on a hot plate. The water was then heated to 55 °C while stirring and the metal precursor was added. Depending on the type of SCG extract being used as the reducing agent, SCG liquid was added to the flask in volumes ranging from 0.5 to 2 mL dropwise as the reaction mixture was stirred with a magnetic stir bar. For the synthesis of silver NPs, 30 mg of solid AgNO_3_ was added to the reaction mixture as the metal precursor. For the synthesis of gold NPs, 0.5 mL of 10mM gold chloride solution was added to the reaction mixture as the metal precursor. Once all reagents were added to the flask, it was constantly stirred for 1 h while maintaining the temperature at 55 °C. The reaction mixture was then cooled naturally to room temperature.

#### 2.6.6. UV-Vis Absorption Spectroscopy

A cuvette was filled with the liquid sample collected, ensuring that no particles of solid coffee grounds were transferred to the cuvette. A Thermo Scientific Evolution 201 spectrophotometer was baselined to water prior to any of the spectra being collected. An absorption spectrum was then collected from 250 to 800 nm for each sample using the UV-vis absorption spectrometer.

#### 2.6.7. SEM Imaging

Scanning electron microscope (SEM) images of samples were taken with a Hitachi FlexSEM 1000. All samples were prepared by centrifuging, washing, and redistributing the silver or gold NPs in deionized water. A droplet from the NP solution was then dried on conducting carbon tape or a carbon-coated, copper mesh transmission electron microscopy (TEM) grid. Analysis of NP size was carried out using the Hitachi FlexSEM software and ImagePro.

#### 2.6.8. Statistical Analysis

Two-way ANOVA analysis with Tukey’s Honest Significant Difference (HSD) test was performed using an R script by Wessa [89]. Differences between means were considered significant at *p* < 0.05.

## 3. Results and Discussion

**Figure 1:**
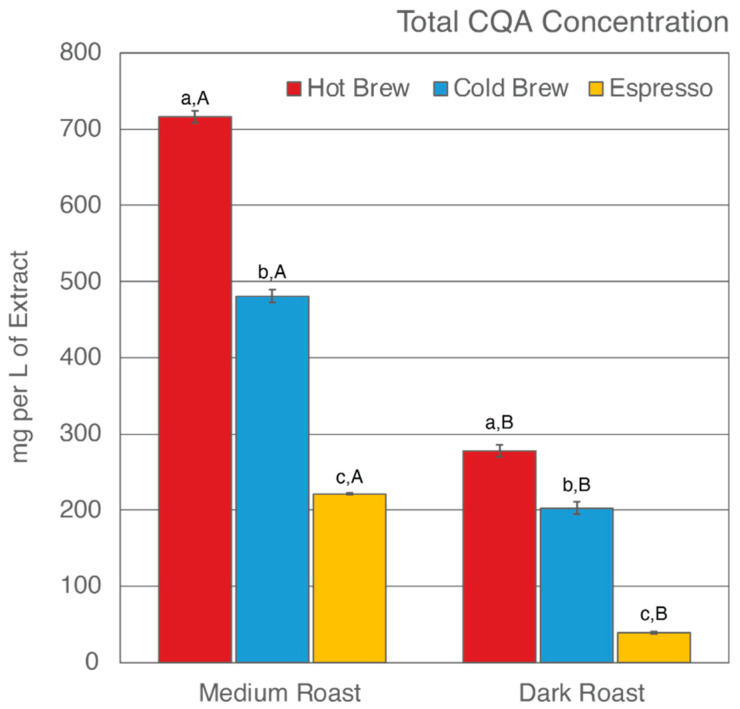
Total CQA concentration of six SCG extracts. Letters a–c denote significant (*p* < 0.05) differences among brewing methods at the same degree of roast as determined by the Tukey HDS post-tests. Letters A and B denote significant differences between medium and dark roast within the same brewing method.

**Figure 2:**
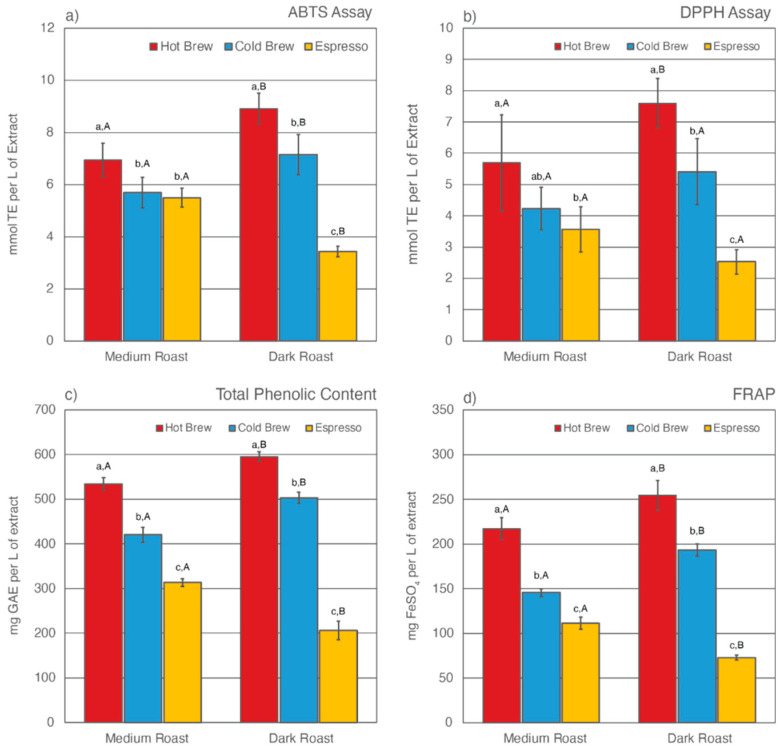
Total antioxidant capacities (TACs) as estimated by ABTS assay (**a**), DPPH assay (**b**), Folin-Ciocalteu assay for total phenolic content (**c**), and ferric ion reducing antioxidant power (**d**) for six SCG extracts. Letters a–c denote significant (*p* < 0.05) differences among brewing methods at the same degree of roast as determined by the Tukey HDS post-tests. Letters A and B denote significant differences between medium and dark roast within the same brewing method.

Previous studies have found that, despite having undergone an initial brewing process, SCGs remain rich in total CQA concentrations and TACs [43,52,54,56,57,58,75,76,77]. Only a few studies have reported how different brewing methods [52,58,59] and degree of roast [53,58] affect what compounds are left behind in SCGs. This study analyzed the total CQA concentrations and TACs of extracts from six SCG samples generated from various brewing methods and degrees of roast. The results are shown in Table 2 and Figure 1 and Figure 2.

### 3.1. Total CQA Concentration

Of the six SCG extracts analyzed, SMH was observed to have the highest total CQA concentration (716.02 mg/L of extract), whereas SDE was observed to have the lowest (38.88 mg/L of extract) (Figure 1). In general, hot brew SCG extracts were observed to have the highest level of CQAs, whereas espresso SCG extracts were observed to have the lowest level of CQAs, regardless of degree of roast. These results suggest that an initial hot brew method did not extract CQAs as efficiently as a cold brew method or espresso brewing, therefore leaving significant CQAs behind in the SCGs. To date, research on the total CQA concentration of SCG extracts from a cold brew method is not available [59]. The cold brew method is known to extract more CQAs due to the prolonged extraction time of the initial brew [46,90,91,92,93]. Indirectly, the observed CQA levels in cold brew SCG extracts were in support of previous findings. The low levels of total CQA concentration in espresso SCG extracts are in agreement with previous studies by Bravo et al. [52]. This finding is corroborated by the high extraction efficiency of the espresso brewing method in comparison to other brewing methods [94,95,96].

Total CQA concentration is also impacted by the degree of roast. It is generally accepted that the roasting process changes the chemical composition of the coffee by producing melanoidin while degrading phenolic compounds [97,98,99]. Numerous studies have reported a decrease in CQA concentration as roasting progresses [82,97,98,100,101]. In this present study, the total CQA concentration was observed to be higher in extracts from medium roast SCG extracts than from dark roast SCG extracts, regardless of which brewing method was used to generate the SCGs (Table 2, Figure 1). The CQA concentration ranged from 221–716 mg/L of extract for medium roast SCGs and 38–277 mg/L of extract for dark roast SCGs. In agreement with previous studies [53,58], the low level of total CQA concentration in dark roast SCG extracts as compared to medium roast SCG extracts supports the understanding that total CQA concentration decreases as degree of roast increases.

### 3.2. Total Antioxidant Capabilities (TAC)

The TAC levels of SCG extracts were estimated using four different methodologies, including ABTS assay, DPPH assay, Folin-Ciocalteu assay for total phenolic content (TPC), and ferric ion reducing antioxidant power (FRAP) (Figure 2). Of the six SCG extracts analyzed, SDH extract exhibited the highest TAC levels as measured by all four methods, whereas SDE extract was observed to have the lowest TAC levels. Similar to total CQA levels, the prolonged extraction time in the cold brew method was responsible for leaving fewer antioxidants behind in the SCGs. As a result, cold brew SCG extracts for both medium and dark roasts had lower TAC levels than their hot brew counterparts. This result contradicts a recent study by Chongsrimsirisakhol and Pirak, in which cold brew SCG extracts were observed to have higher TAC levels than hot brew SCG extracts [59]. This discrepancy may be attributed to differences in the coffee-to-water ratio, brewing time, water temperature, and bean origin.

The degree of roast also affects TAC levels in SCG extracts. For both hot and cold brewing methods, dark roast SCG extracts were observed to have higher TAC levels than medium roast counterparts. This trend was reversed for espresso brewing methods, in which SME extract was observed to have higher TAC levels than SDE extract. Previous studies have reported that the formation of melanoidins during roasting helps to stabilize TAC levels in coffee [98,99,101]. These complex melanoidin compounds are more soluble in hot water than cold water [82,93,102,103,104]. Although the water temperature in hot brew methods was high enough to extract some of the additional antioxidants formed during roast, the short brewing time significantly lowered the extraction efficiency of the hot brew method, leaving plenty of antioxidants behind in the SCGs. As a result, SDH extract had the highest TAC levels compared to other SCG extracts.

The TAC levels of espresso SCG extracts have been reported in multiple previous studies [52,56,58,59,75]. It is generally accepted that espresso SCGs are rich in phenolic compounds. However, there has been no research on how degree of roast affects TAC levels in espresso SCG extracts. The espresso brewing method differs drastically from cold or hot brew methods. In addition to high water temperature, espresso extraction is performed under high pressure using finely ground coffee [105,106,107,108]. Cruz et al. noted a high variability in the chemical composition of espresso SCGs and suggested that “the brewing method itself should be the main contributing factor to the compositional variance” [54].

### 3.3. Green Synthesis of Nanoparticles

**Figure 3:**
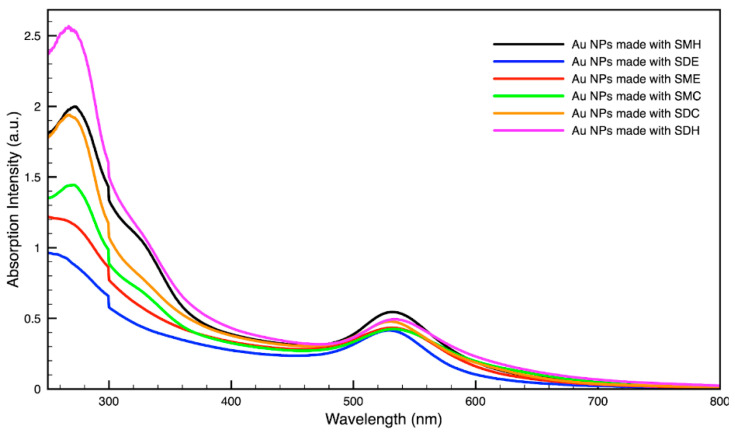
Absorption spectrum of gold (Au) nanoparticles (NPs) synthesized using various forms of spent coffee ground extract.

**Figure 4:**
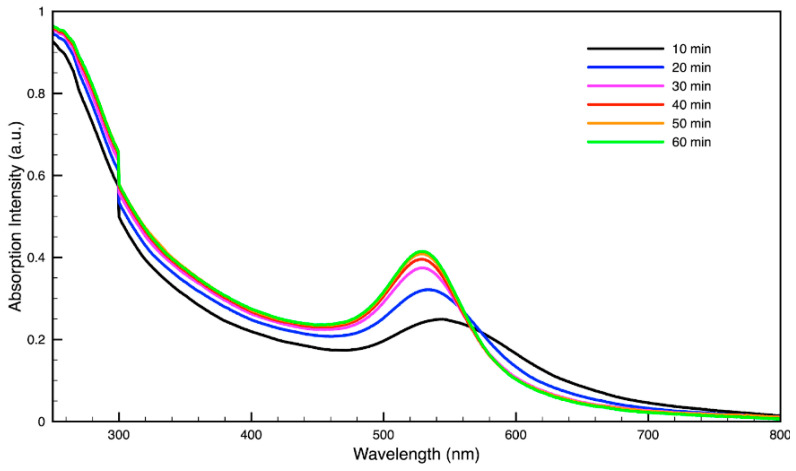
Time evolution of absorption spectrum of gold (Au) nanoparticles during synthesis using dark roast, spent espresso coffee grounds (SDE).

**Figure 5:**
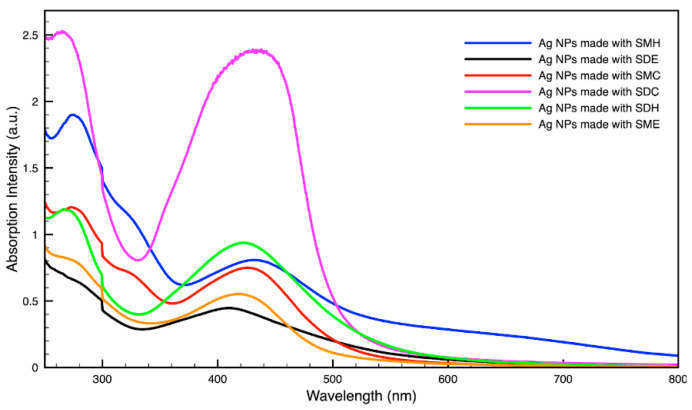
Absorption spectrum of silver (Ag) nanoparticles (NPs) synthesized using various forms of spent coffee ground extract.

**Figure 6:**
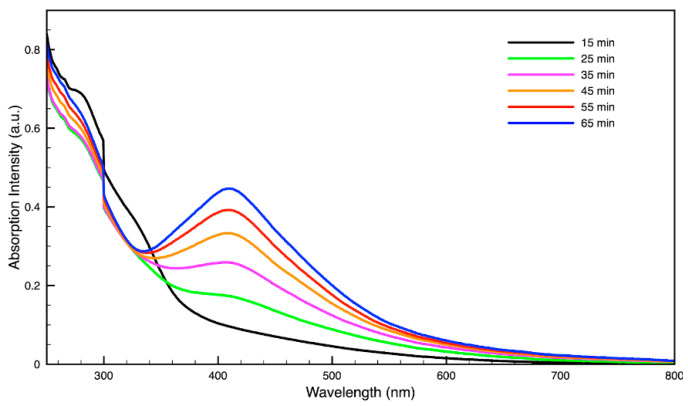
Time evolution of absorption spectrum of silver (Ag) nanoparticles during synthesis using dark roast, spent espresso coffee grounds (SDE).

**Figure 7:**
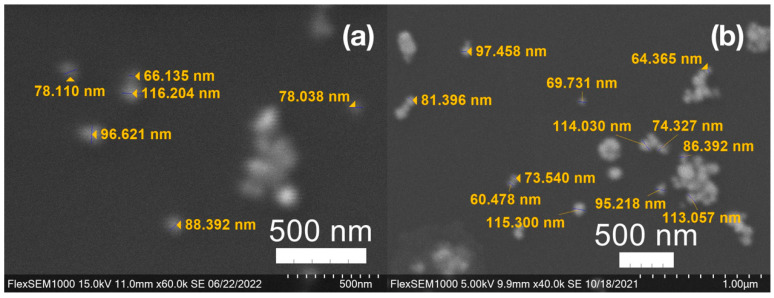
(**a**) Gold (Au) and (**b**) silver (Ag) nanoparticles synthesized with medium roast coffee grounds (SCGs) from French press brew (SMH).

**Figure 8:**
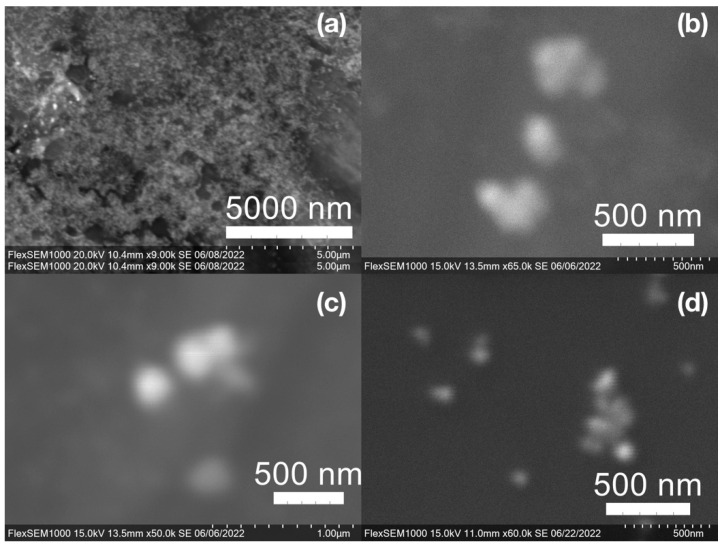
Gold (Au) nanoparticles synthesized with (**a**) medium roast coffee grounds (SCGs) from French press brew (SMH); (**b**,**c**) dark roast SCGs from espresso brew (SDE); and (**d**) medium roast SCGs from cold brew (SMC).

**Figure 9:**
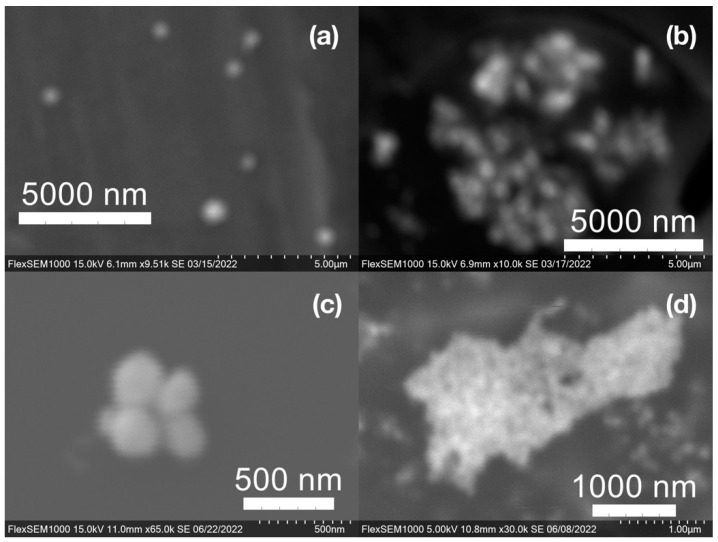
Silver (Ag) nanoparticles synthesized with (**a**) medium roast coffee grounds (SCGs) from French press brew (SMH); (**b**,**c**) medium roast SCGs from cold brew (SMC); and (**d**) dark roast SCGs from espresso brew (SDE).

**Figure 10:**
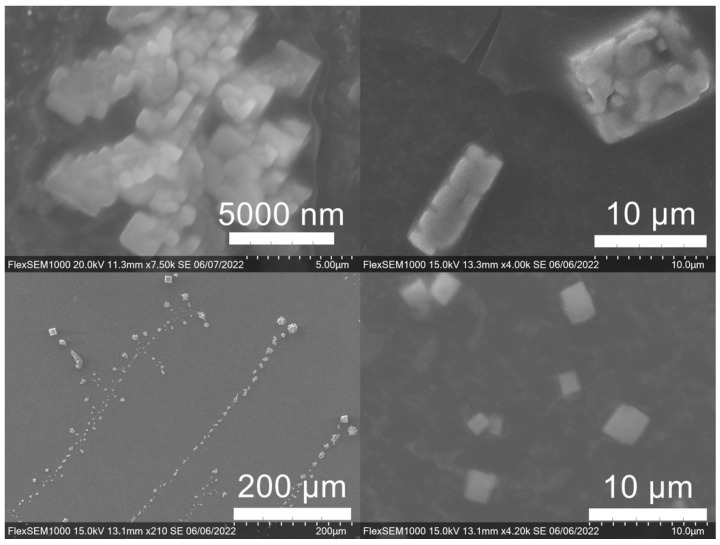
Gold (Au) nanoparticles synthesized with dark roast coffee grounds from espresso brew (SDE) forming larger superstructure assemblies.

**Figure 11:**
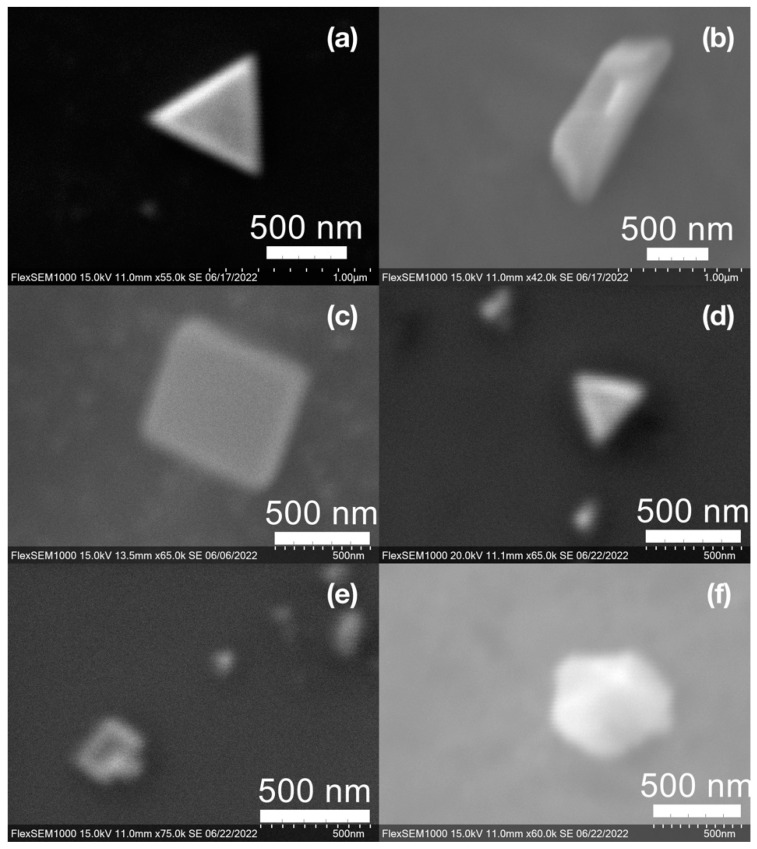
Various gold (Au) nanoparticle shapes synthesized using spent coffee grounds of (**a**–**c**) dark roast used in espresso (SDE), and (**d**–**f**) medium roast used in cold brew coffee (SMC).

**Figure 12:**
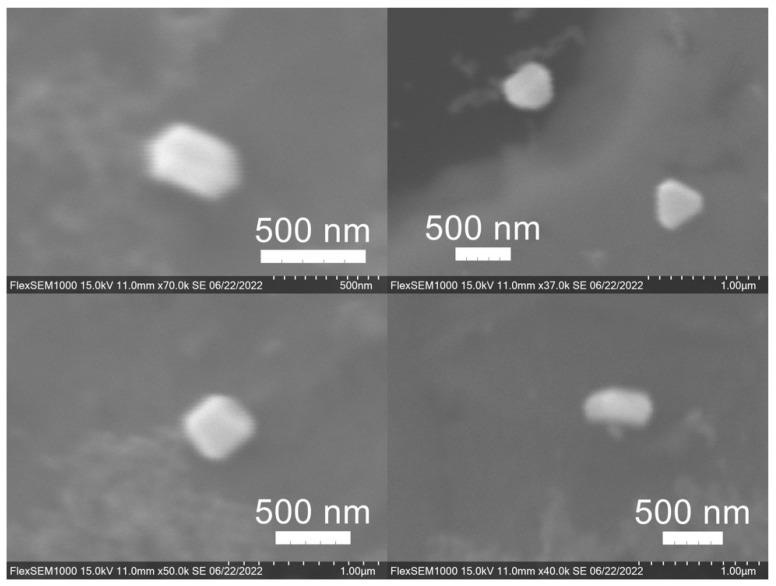
Silver (Ag) nanoparticles synthesized using spent coffee grounds of medium roast used in cold brew coffee (SME).

For metal nanoparticles (NPs), it is well known that the absorption of specific wavelengths of light is correlated to the size and shape of the NPs through the effect of the surface plasmon resonance (SPR) [109,110,111]. Therefore, taking an absorption spectrum of the NPs in solution can reveal a great deal of information about the NPs. In the case of gold NPs synthesized using SCGs, a strong absorption peak was observed in the green region of the visible spectrum (about 540 nm) at the start of the synthesis. As the synthesis continued, the peak shifted towards the blue region to end between 525–535 nm (Figure 3 and Figure 4). An absorption peak at these wavelengths indicates an average diameter between 10–80 nm for spherical Au NPs [111,112,113] and an edge length of 15–50 nm for Au nanocubes [114]. As is typical for a single-pot, benchtop NP synthesis, a range of sizes and shapes were found in each sample and confirmed by SEM (Figures 7–12). While different shapes may cause additional absorption peaks further in the red and infrared regions, all Au NPs will exhibit a characteristic SPR band between 500–550 nm [111,112]. Additionally, the relative broadness of the absorption bands indicates a wide distribution of NP sizes present in each sample. In the case of silver NPs, an absorption peak was seen to develop in the 410–455 nm range initially, then became stronger throughout the synthesis without much shift in the peak absorption wavelength (Figure 5 and Figure 6). An absorption peak at these wavelengths for silver NPs indicates a size ranging between 20–100 nm [110,115,116,117,118]. Similar to Au NPs, all Ag NPs will exhibit a characteristic SPR band between 400–450 nm with some nonspherical shapes contributing additional absorption peaks in the red and infrared regions [112,117].

SEM images of the gold NP samples indicate that many of the individual NPs range in diameter from 66–120 nm (Figure 7a), although the exact size is difficult to determine due to the resolution of the imaging system. NPs with dimensions in the 100 s of nanometers were also observed (Figure 8, Figure 9, Figure 10 and Figure 11). Larger clusters of NPs can also be seen (Figure 8 and Figure 10), which is not unusual in aqueous benchtop synthesis methods without the addition of a stabilizing agent to prevent NPs from sticking together. Images of the silver NP samples also indicate a range of individual NP dimensions from 60–120 nm for the smaller ones (Figure 7b) and in the 100s of nanometers (Figure 12) for larger particles. In the gold NP samples, larger cubic superstructures comprised of individual NPs were also observed (Figure 10), which suggests that many of the individual NPs are in fact nanocubes [119,120,121], with the superstructures resembling a Rubix cube. Other shapes were observed in the gold NP samples including pyramids, trapezoids, and spheres (Figure 11) with features ranging in size from 30–500 nm. Silver NP samples also had a variety of shapes (Figure 12) including prisms, hexagons, plates, and cubes with dimensions ranging from 30-400 nm. The presence of a wide range of sizes and shapes is an indication of reduction of metal ions and unchecked growth of NPs throughout the synthesis phase due to the strong antioxidant activity of the SCG extract. Once NPs are formed in an aqueous solution, any remaining reducing agents may cause NPs to continue to grow in size, agglomerate into multiparticle clusters, and fuse into micron-sized structures [111,112,113].

Comparing the silver and gold NP samples made with the same amount of metal precursor and extract from SCGs, some variation in peak absorption wavelength can be observed (Table 3), which indicates differentiation in sizes and shapes of NPs present. These differences may be due to more complex interactions between the various active compounds, as well as the physical chemistry involved in growing and shaping the NPs in these particular environments. SDE and SME, which had the lowest TAC levels, produced silver NPs with the lowest SPR absorption wavelength, indicating that the average NP size was smaller when using espresso SCG extract as a reducing agent. However, this was the only discernible correlation between the peak absorption wavelength and TAC or CQA levels present in the SCG extract.

**Figure 13:**
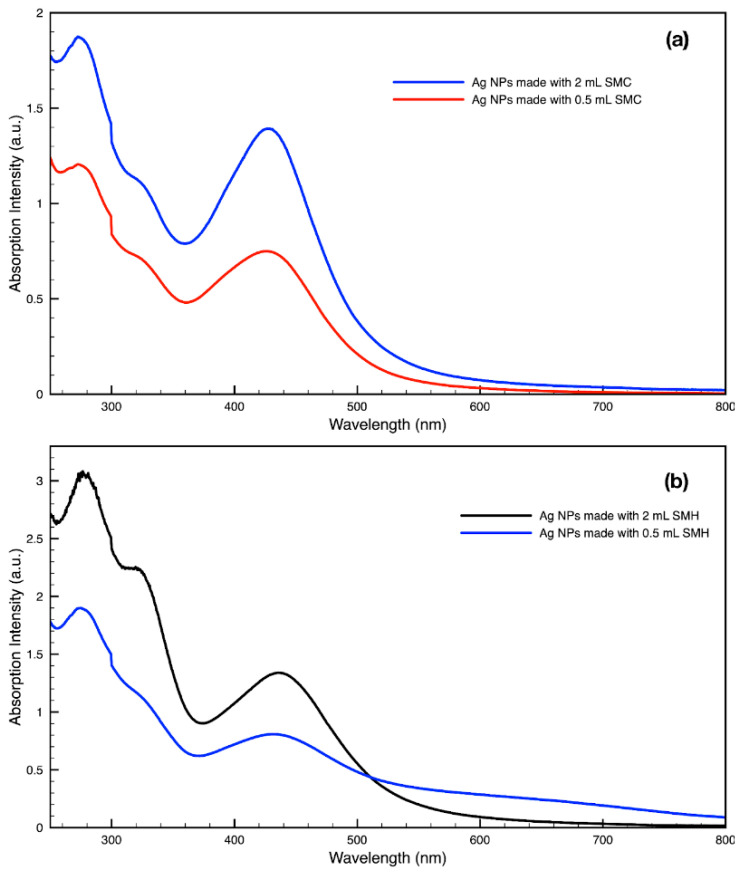
Absorption spectra of silver (Ag) nanoparticles (NPs) synthesized with either 2 mL or 0.5 mL of (**a**) SMC extract or (**b**) SMH extract per 30 mL sample.

**Figure 14:**
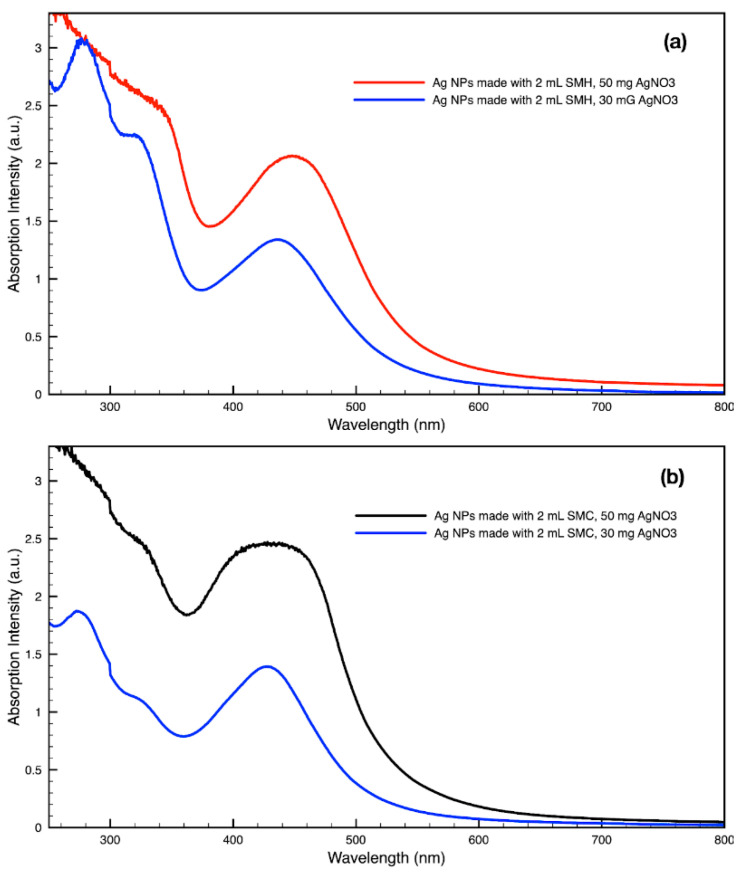
Absorption spectra of silver (Ag) nanoparticles (NPs) synthesized with either 30 mg or 50 mg of metal precursor, AgNO_3_, per 30 mL sample by (**a**) SMH and (**b**) SMC.

By varying the ratio of SCG extract to the total liquid volume while synthesizing NPs, it was seen that more SCG extract resulted in a more intense absorption peak without changing the peak wavelength (Figure 13). This suggests that within the range explored in this work, 0.5–2 mL, the addition of more SCG extract will yield more NPs without altering their relative sizes and shapes. By contrast, varying the ratio of metal precursor to total liquid volume corresponded to a shift in the peak absorbance wavelength (Figure 14). Samples synthesized using extract from SMH with more metal precursor yielded NPs with a peak wavelength of 449 nm, while the batch with less metal precursor had a peak wavelength of 437 nm. Samples synthesized using extract from SMC with more metal precursor yielded NPs with a peak wavelength of 434 nm, while the batch with less metal precursor had a peak wavelength of 428 nm. Since longer absorption wavelengths are correlated to larger size in metal NPs [112], these results indicate that higher metal precursor concentrations yield larger NPs overall rather than more NPs of the same size. More research is needed to investigate a wider range of metal precursor concentrations. Future studies should also clarify the exact roles the chemical components in SCG extracts play in reducing metal ions, promoting NP aggregation and growth, preferentially growing specific NP shapes, and capping or surface coating these NP species. Various metal, oxide, and other species of NPs which are synthesized by similar reduction methods can likely use this SCG extract technique.

## 4. Conclusions

The chemical components present in spent coffee grounds (SCG) available for ex-traction through a simple secondary brew process were characterized and quantified including total caffeoylquinic acid (CQA), total antioxidant capacities (TACs), as well as efficacy in synthesizing gold and silver nanoparticles (NPs). SCGs of both medium and dark roasts, which were initially brewed using hot brew, cold brew, and espresso methods, were investigated. It was found that SCGs from an initial hot brew exhibited the highest CQA and TAC levels, followed by SCGs from an initial cold brew, with SCGs from an initial espresso brew exhibiting the lowest CQA and TAC levels regardless of degree of roast. Compared to dark roast SCGs, medium roast SCGs yielded higher CQA levels but lower TAC levels whether processed via hot or cold brewing methods. This trend was reversed for espresso-derived SCGs, which showed higher CQA levels and lower TAC levels for the dark roast. This work also demonstrated a green chemistry, single-pot, aqueous workflow for synthesizing both gold and silver NPs with only two reagents, SCG extract and a metal precursor. It is shown that silver and gold NPs of various shapes and sizes ranging from 10 s of nm to 500 nm may be easily synthesized using SCGs regardless of the initial brewing method or degree of roast. Therefore, SCGs are shown to be as effective as coffee bean extract, coffee liquid, and fresh coffee grounds in synthesizing metal NPs through green chemistry methods. This confirms the utility of food-waste materials such as SCGs in synthesizing nanomaterials, whose wide-ranging applications provide a value-added opportunity to sectors of the economy that produce or handle SCGs.

## Figures and Tables

**Table 1:** Sample Code by Variation in Preparation.

SCG Sample Code	Roast	Brewing Method Used to Generate the SCG
SMC	Medium	Cold brew; the particle size of the coffee grounds ranged from 710 µm to 1000 µm; the grounds were steeped in 22 °C deionized (DI) water for 24 h.
SMH	Medium	Hot brew; the particle size of the coffee grounds ranged from 710 µm to 1000 µm; the grounds were steeped in 100 °C DI water for 6 min.
SME	Medium	Espresso brew; the particle size of the coffee grounds was below 500 µm.
SDC	Dark	Cold brew; the particle size of the coffee grounds ranged from 710 µm to 1000 µm; the grounds were steeped in 22 °C DI water for 24 h.
SDH	Dark	Hot brew; the particle size of the coffee grounds ranged from 710 µm to 1000 µm; the grounds were steeped in 100 °C DI water for 6 min.
SDE	Dark	Espresso brew; the particle size of the coffee grounds was below 500 µm.

**Table 2:** Total CQA Concentration and TAC Levels of Six (6) SCG Extracts.

SCG Samples	Total CQA Concentration (mg/L of Extract)	ABTS (mmol TE/L Extract)	DPPH (mmol TE/L Extract)	TPC (mg GAE/L Extract)	FRAP (mg FeSO_4_/L Extract)
SMC	480.65 ± 8.31 ^a,A^	5.69 ± 0.59 ^a,A^	4.23 ± 0.68 ^a,A^	420.5 ± 16.7 ^a,A^	145.6 ± 4.2 ^a,A^
SMH	716.02 ± 7.70 ^b,A^	6.94 ± 0.64 ^b,A^	5.69 ± 1.54 ^ab,A^	534.2 ± 14.0 ^b,A^	217.1 ± 12.3 ^b,A^
SME	221.12 ± 1.17 ^c,A^	5.5 ± 0.37 ^b,A^	3.57 ± 0.72 ^b,A^	313.5 ± 8.9 ^c,A^	111.4 ± 6.8 ^c,A^
SDC	202.72 ± 3.67 ^a,B^	7.15 ± 0.78 ^a,B^	5.41 ± 1.05 ^a,B^	503.2 ± 12.6 ^a,B^	193.3 ± 6.9 ^a,B^
SDH	277.86 ± 4.47 ^b,B^	8.92 ± 0.59 ^b,B^	7.6 ± 0.79 ^b,A^	595.2 ± 11.0 ^b,B^	254.6 ± 16.6 ^b,B^
SDE	38.88 ± 0.71 ^c,B^	3.43 ± 0.20 ^c,B^	2.53 ± 0.39 ^c,A^	206.2 ± 21.1 ^c^	73.1 ± 2.7 ^c,B^

Values are reported as mean ± SD with n = 6. The superscripts a–c denote significant (*p* < 0.05) differences among brewing methods at the same degree of roast as determined by the Tukey HDS post-tests. The superscripts A and B denote significant differences between medium and dark roast within the same brewing method.

**Table 3:** Peak absorption wavelength of nanoparticles (NPs) prepared with 0.5 mL of spent coffee ground (SCG)-derived extract per 30 mL sample.

SCG Samples	Peak Wavelength of Silver NPs	Peak Wavelength of Gold NPs
SMC	427 nm	533 nm
SMH	432 nm	532 nm
SME	419 nm	532 nm
SDC	433 nm	530 nm
SDH	423 nm	534 nm
SDE	410 nm	529 nm

## Data Availability

Not applicable.
